# New Publications

**Published:** 1898-12

**Authors:** 


### NEW PUBLICATIONS.

PHYSICIANS' VISITING LIST FOR 1899. P. Blakiston's Son & Co., Phila.

THIS well-known *Visiting List* appears for 1899 as the forty-eighth yearly edition. No testimonial as to its worth and merit can be more pointed than this, as it indicates its appreciation among a large number who yearly have had it in use. The many valuable tables it contains for ready reference have been constantly added to and more and more relied upon by the busy practitioner. Many veterinarians have found it quite convenient for their use, and it will no doubt find a wider field of employment in this direction. As the profession becomes better acquainted with the ready manner it affords one of rendering a requested account it will find increased appreciation of its merits.

THE Pope Manufacturing Company has sent forth its Annual Calendar for 1899, in which the merits of the Columbia Wheel are brought out more prominently than ever. Its value on one's office desk as a daily reminder of engagements fills a useful purpose for a busy man, and its many apt quotations are interesting and instructive. Colonel Pope, the head of this corporation, will be best remembered by the veterinary profession for his earnest, aggressive, and successful agitation for good roads, which, while contributing to the increased use of the bicycle, has at the same time added thousands to the great numbers constituting the driving public.

---

Dr. William H. Yingst, of Harrisburg, Pa., a volunteer in the army and who was assigned to veterinary duties while in the service, was mustered out early in November. He has no desire to return to army life under present conditions. The doctor says that in order to partially discharge his professional duties to his patients he expended from his own funds sixteen dollars for medicines, which expenditure was rendered necessary owing to the inadequate and antiquated supplies furnished for the army veterinary service.

---

Dr. Leonard Pearson, State Veterinarian of Pennsylvania, was a recent visitor to Sing Sing, N. Y.

